# Evaluation of Neurocognitive Functions of Children and Adolescents Diagnosed with Pediatric Bipolar Disorder and Disruptive Mood Dysregulation and at High Risk for Bipolar Disorder

**DOI:** 10.1192/j.eurpsy.2024.207

**Published:** 2024-08-27

**Authors:** I. Yenen Sivri, E. Çöp, K. C. Can, A. S. Yenen Menderes

**Affiliations:** ^1^Child and Adolescent Psychiatry, Osmaniye State Hospital; ^2^Child and Adolescent Psychiatry; ^3^Adult Psychiatry, Ankara City Hospital; ^4^Child and Adolescent Psychiatry, Keçiören Educational Research Hospital, Ankara, Türkiye

## Abstract

**Introduction:**

Pediatric Bipolar Disorder (BPD) is a chronic psychiatric disorder that alters normal and psychological development processes among patients. Although cognitive deficits in BPD have identified in recent studies, little is known about the developmental trajectory of these deficits. DMDD is a newly defined diagnosis included in the DSM-V. Since it added a new dimension to the clinical spectrum but few studies conducted on DMDD, there are some conflicting discussions in the literature about how to distinguish this disorder from other childhood psychiatric disorders and how to treat it.

**Objectives:**

The aim of this study was to determine the phenomenological and neuropsychological differences between children and adolescents with a diagnosis of BPD (Pediatric Bipolar Disorder), DMDD (Disruptive Mood Dysregulation Disorder), and children and adolescents who are genetically at high risk for Bipolar Disorder (BD), and healthy controls (HCs) who do not have any psychiatric diagnosis, to investigate endophenotypes that may be predictive for BD.

**Methods:**

Our study sample consists of four groups, the BPD group (n=30), the Risk group (n=25), the DMDD group (n=36), and the Healthy Control group (n=29). All participants were evaluated by the “Kiddie Schedule for Affective Disorders and Schizophrenia for School-Age Children—Now and Lifetime Pattern (K-SADS-PL)”. “Young Mania Rating Scale/Parent Form (YMRS-ABF), Conner’s Parent Rating Scale (CPRS-48), Child and Adolescent Behavior Rating Scale (CBCL)” scales were filled by parents, and “Child Depression Inventory (CDI), Youth Self-Report Form for 11-18 Years Olds (YSR)” scales were filled by children and adolescents. Neurocognitive test battery was applied to each participant: Continuous Performance Test (CPT), Wisconsin Card Sorting Test (WCST), Stroop Color and Word Test (SCWT), Trait Making Test A and B sections (TMT-A/B), California Verbal Learning Test-Child version (CVLT-C).

**Results:**

While it was determined that the cases in the BPD and DMDD groups performed significantly worse in CPT, SCWT, CVLT-C, TMT A/B tests compared to healthy controls, it was found that the subjects in the Risk group performed worse at the CPT test than healthy controls. In addition, the cases in the BPD, Risk and DMDD groups reported more clinical and behavioral problems than the healthy controls.

**Conclusions:**

There is a significant deterioration in the areas of continuous attention, processing speed, cognitive flexibility, response prevention, verbal memory and working memory in the BPD and DMDD groups, and in the continuous attention area in the Risk group compared to healthy controls. Prospective follow-up and imaging studies using larger samples and a larger neurocognitive test battery in the future will better reveal the neuropsychological characteristics of the BPD, Risk and DMDD groups.

**Disclosure of Interest:**

None Declared

